# Dissecting the Roles of the Cytokinin Signaling Network: The Case of De Novo Shoot Apical Meristem Formation

**DOI:** 10.3390/biom14030381

**Published:** 2024-03-21

**Authors:** Nina Pokimica, Tatjana Ćosić, Branka Uzelac, Slavica Ninković, Martin Raspor

**Affiliations:** Department of Plant Physiology, Institute for Biological Research “Siniša Stanković”—National Institute of Republic of Serbia, University of Belgrade, Bulevar Despota Stefana 142, 11060 Belgrade, Serbia; tatjana@ibiss.bg.ac.rs (T.Ć.); branka@ibiss.bg.ac.rs (B.U.); slavica@ibiss.bg.ac.rs (S.N.)

**Keywords:** cytokinin, de novo shoot organogenesis, histidine kinase, histidine phosphotransfer protein, response regulator, shoot apical meristem, shoot regeneration, signal transduction, SHOOTMERISTEMLESS, WUSCHEL

## Abstract

Numerous biotechnological applications require a fast and efficient clonal propagation of whole plants under controlled laboratory conditions. For most plant species, the de novo regeneration of shoots from the cuttings of various plant organs can be obtained on nutrient media supplemented with plant hormones, auxin and cytokinin. While auxin is needed during the early stages of the process that include the establishment of pluripotent primordia and the subsequent acquisition of organogenic competence, cytokinin-supplemented media are required to induce these primordia to differentiate into developing shoots. The perception of cytokinin through the receptor ARABIDOPSIS HISTIDINE KINASE4 (AHK4) is crucial for the activation of the two main regulators of the establishment and maintenance of shoot apical meristems (SAMs): *SHOOTMERISTEMLESS* (*STM*) and the *WUSCHEL-CLAVATA3* (*WUS-CLV3*) regulatory circuit. In this review, we summarize the current knowledge of the roles of the cytokinin signaling cascade in the perception and transduction of signals that are crucial for the de novo establishment of SAMs and lead to the desired biotechnological output—adventitious shoot multiplication. We highlight the functional differences between individual members of the multigene families involved in cytokinin signal transduction, and demonstrate how complex genetic regulation can be achieved through functional specialization of individual gene family members.

## 1. Introduction

Auxins and cytokinins are the most important phytohormones responsible for the regulation of plant growth and development, including cell division and differentiation, root and shoot meristem formation, apical dominance, senescence, cell metabolism, morphogenesis and numerous other physiological processes in plants [1].

The essential role of auxin and cytokinin has also been observed in the process of in vitro regeneration of shoots from explants originating from different plant organs when nutrient media are enriched with these hormones in a precisely determined balance of concentrations. This process, which is extensively used for the clonal propagation of whole plants in numerous biotechnological applications, is known as de novo shoot organogenesis (DNSO) [2]. Shoot primordia can develop directly on the surface of explants without the occurrence of a callus [3,4,5,6,7,8,9], but more often, shoot regeneration proceeds through a stage of an unorganized cell mass—the callus—in a process termed indirect shoot organogenesis. The classic protocol involves growing explants in vitro commonly using two types of media with different ratios of auxin and cytokinin concentrations. Namely, a callus induction medium (CIM), which contains high auxin and low cytokinin concentrations, is used first, followed by a shoot induction medium (SIM) which is characterized by high concentrations of cytokinin and low auxin content [10]. Skoog and Miller [11] showed that the ratio of auxin-to-cytokinin concentrations determines the further morphogenetic pathway and the formation of different plant organs. A higher auxin-to-cytokinin ratio stimulates the formation of roots, while conversely, a higher concentration of cytokinin compared to auxin promotes shoot growth. Accordingly, CIM is necessary for the auxin-dependent development of pluripotent primordia within the incubated explant, whereas subsequent incubation on SIM ensures the cytokinin-dependent differentiation of these pluripotent primordia into shoot meristems which are necessary for shoot regeneration [12].

Although indirect shoot organogenesis has been used in biotechnology for several decades, some important features of the underlying processes have been revealed only over a decade ago. In 2009, Atta and co-authors [3] found that the incubation of root or hypocotyl explants of *Arabidopsis thaliana* on CIM leads to the formation of “protuberances” within the pluripotent callus. These protuberances, which originated within the xylem pole of the pericycle in the plant organs used as explants, bore striking resemblance to lateral root meristems, regardless of whether they were induced in the root or hypocotyl explants. Moreover, their gene expression profiles corresponded to the gene expression profiles of lateral root meristems. However, upon transfer to SIM, these primordia, which resemble the lateral root primordia (LRP), increased in size, turned green and started expressing marker genes of the shoot apical meristem (SAM): *SHOOTMERISTEMLESS* (*STM*), *WUSCHEL* (*WUS*) and *CLAVATA3* (*CLV3*). These results suggested that the organogenic callus is not an entirely homogenous, disorganized tissue; on the contrary, it contains organogenic primordia that are responsive to phytohormones and that, upon cytokinin treatment, are able to differentiate into shoots [3].

These findings were corroborated by Sugimoto and co-authors [13], who confirmed that, in the early phases of indirect shoot organogenesis, even the calli induced from aerial organs such as cotyledons or petals, contained structural elements similar to LRP. The broad expression of *J0121*, a genetic marker of the xylem pole-associated pericycle cells, throughout the callus confirmed that the growing mass of callus cells originated from the xylem pole of the pericycle. In addition, homozygous *aberrant lateral root formation4* (*alf4*) mutants, which are deficient in pericycle cell division and lateral root formation, were also unable to form calli on the CIM, and consequently, to regenerate shoots in response to cytokinin treatment on the SIM. These results confirmed that pericycle-like cells that are present around vascular elements throughout the plant body underlie the pluripotency of an array of plant tissues from which shoot meristems can be regenerated [13].

Upon the incubation of explants on the CIM, the auxin from the regeneration medium induces the specification of founder cells from which the organogenic primordia will later arise. Founder cells are dependent on the establishment of local auxin maxima in pericycle or pericycle-like cells. This process is not possible without the simultaneous spatiotemporally regulated expression of genes coding for auxin transporters: first, AUX1 transporters that ensure auxin influx into cells, and PIN-FORMED (PIN) transporters that enable auxin efflux from cells [14]. Thus, these transporters mediate the establishment of a dynamic auxin gradient. In order to establish local auxin maxima that will consequently lead to the specification of founder cells, the positive regulation of the *AUX1* gene, as well as the negative regulation of the *PIN* genes are necessary to allow for the influx and prevent the efflux of auxin from the cells [15].

The formation of local auxin maxima is directly followed by the expression of genes involved in the regulation of primordia formation, belonging to the *WUSCHEL-RELATED HOMEOBOX* (*WOX*), *LATERAL ORGAN BOUNDARIES DOMAIN* (*LBD*) and *PLETHORA* (*PLT*) gene families [16,17]. Sugimoto and co-authors reported that, even when aerial organs were used as explants for the induction of a pluripotent callus during the early phases of shoot organogenesis, gene expression patterns typical of root primordia were observed, including the expression of *SCARECROW* (*SCR*), *SHORTROOT* (*SHR*), *GLABRA2* (*GL2*) and *WOX5* [13]. Specifically, *WOX5* is known as a regulator of pluripotency and cell proliferation in the root stem cell niche [18]; thus, it was assumed to perform an equivalent role in the maintenance of pluripotency of the primordia within the organogenic callus. Furthermore, *LBD* is a large family of auxin-activated transcription factors with a broad spectrum of metabolic and morphological outputs resulting in the differentiation, development and metabolism of the pluripotent primordium [16,19,20]. Conversely, *PLT* is a family of transcription factors that are positioned at the center of the gene regulatory networks that govern all subsequent stages of organogenesis, making them essential master regulators of DNSO [21]. As such, *PLT3*, *PLT5* and *PLT7* were found to be partially functionally redundant in the organogenic response, with triple loss-of-function *plt3plt5plt7* mutants completely unable to regenerate shoots [22].

After the pluripotent primordia develop under the influence of exogenous (or endogenous) auxin, the subsequent process of acquisition of competence for organogenesis is regulated by the *PLT*, *ENHANCER OF SHOOT REGENERATION* (*ESR*) and *CUP-SHAPED COTYLEDON* (*CUC*) gene families, and requires initial incubation on the CIM, but the subsequent incubation on the SIM is also necessary, since this stage of organogenesis is regulated by both auxins and cytokinins [21,22,23,24]. This phase involves a change in the identity of the pluripotent primordia, whereby the *WOX5*, *PLT1* and *SHR* expression is silenced under the influence of cytokinin and replaced by a gene expression pattern typical of the shoot promeristem tissue, causing the primordia to lose their root-like phenotype and acquire the identity of shoot promeristems [4,5].

Contrary to a two-step protocol where a serial application of the CIM and SIM is required, de novo shoot organogenesis in some species can be obtained on a single regeneration medium, corresponding to the SIM [12]. The ability of plant tissues to undergo complete shoot regeneration on a single SIM medium was suggested to depend on their endogenous phytohormone content. In kohlrabi (*Brassica oleracea* var. *gongylodes*), seedling explants, which were characterized by high endogenous auxin-to-cytokinin ratios, showed greater shoot regeneration efficiency on the SIM than hypocotyl explants that were more rich in cytokinin [25]. Over the course of shoot regeneration, dynamic and complex changes were observed in the kohlrabi phytohormonome, suggesting that the process of shoot regeneration extensively interacts with the metabolism of all plant hormones [26]. The incubation of seedling explants on a cytokinin-rich SIM caused an increase in endogenous auxin levels that was even greater than the increase in endogenous cytokinin, resulting in a net increase in the endogenous auxin-to-cytokinin ratio, but also in increased shoot regeneration efficiency. Thus, the ability of kohlrabi seedlings to undergo complete regeneration on SIM seems to depend on the effects of SIM on their endogenous auxin metabolism, which are equivalent to exogenous auxin supplementation in the early phases of organogenesis [25]. The expression profiles of selected cell cycle- and organogenesis-related genes suggested that during the one-step shoot regeneration of kohlrabi, the explants underwent the same series of developmental events as in the two-step regeneration from *Arabidopsis* root explants, confirming the universal nature of the shoot regeneration mechanism across plant species [27].

The last phase of organogenesis is regulated by the target elements of the cytokinin signaling cascade, *SHOOTMERISTEMLESS* (*STM*), *WUSCHEL* (*WUS*) and *CLAVATA3* (*CLV3*). The onset of the expression of *STM* and the *WUS-CLV3* circuit marks the beginning of shoot apical meristem (SAM) formation [4,5]. In this review, we summarize the current insights on how the elements of the cytokinin signaling cascade regulate shoot regeneration through their interplay with these two key regulators of the SAM, in *A. thaliana* as the main model, as well as in other researched model plants. We emphasize that, although cytokinin signaling relies on perception and signal transduction that involve multi-member families of receptors and response regulators, individual members of these seemingly functionally redundant gene families actually play distinct regulatory roles, resulting in an extremely complex and finely tuned regulatory network that controls the de novo formation of shoot meristems.

## 2. Genetic Regulation of Shoot Apical Meristem (SAM) Formation

**Figure 1:**
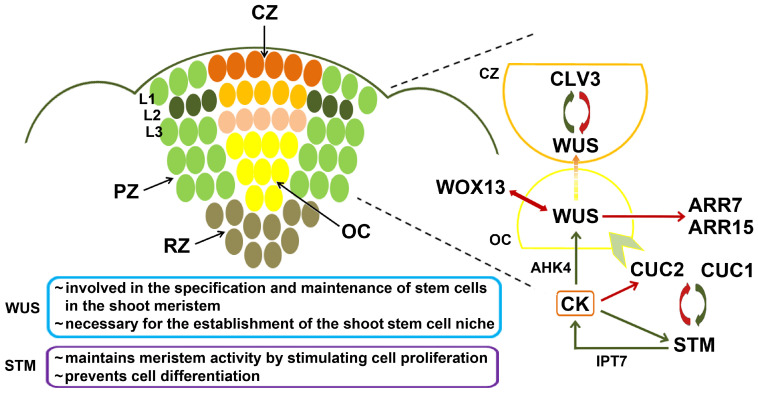
Shoot apical meristem (SAM) zones in *Arabidopsis thaliana*. The central zone (CZ) is organized in specific layers L1, L2 and L3, and contains cells with low mitotic activity. The organizing center (OC) is positioned below the CZ and is characterized by a constant number of stem cells. The OC is surrounded by the peripheral zone (PZ) consisting of rapidly dividing cells, and the rib zone (RZ). The right-hand part of the diagram represents an “enlargement” of the left-hand part, summarizing the regulatory interactions characteristic of the CZ (orange) and the OC (yellow). The key genes involved in SAM formation are *WUSCHEL* (*WUS*) and *SHOOTMERISTEMLESS* (*STM*). Under the positive influence of cytokinin (CK) signaling through the AHK4 receptor, *WUS* is expressed in the OC, and its product further migrates to the CZ, where it positively regulates the expression of *CLAVATA3* (*CLV3*). The CLV3 peptide inhibits *WUS* outside of the OC, thereby establishing a negative feedback loop. *WUS* promotes CK signaling also by repressing the negative regulators of the CK response, *ARABIDOPSIS RESPONSE REGULATOR7* (*ARR7*) and *ARR15*. Transcription factors CUP-SHAPED COTYLEDON1 (CUC1) and CUC2 stimulate *STM* activity, which in turn represses *CUC1* and *CUC2*, indicating the existence of a negative feedback loop. *STM* is positively regulated by CK and vice versa. STM itself positively regulates CK biosynthesis through the activation of *ISOPENTENYL-TRANSFERASE7* (*IPT7*). Green arrows indicate positive regulation, whereas red arrows indicate negative regulation. The temporal succession of morphogene expression in the area of shoot progenitors where the expression of *CUC2* is replaced with *WUS* expression is represented by a pale green chevron arrow.

The shoot apical meristem (SAM) is formed during zygotic embryo development and is involved in the formation of the stem, leaves, reproductive and other aboveground organs, and it also plays a significant role in maintaining the pool of meristem cells throughout the life of a plant. It consists of several zones (Figure 1): the central zone (CZ) comprising cells with low mitotic activity, the peripheral zone (PZ) with relatively fast-dividing cells, and the rib zone (RZ) that provides cells for the internodes. The slow cell divisions in the CZ gradually push some cells toward the PZ where they differentiate, leading to the initiation of lateral organ primordia [28,29,30]. Below the CZ lies the organizing center (OC) in which a constant number of stem cells is maintained [31,32,33,34,35]. The CZ of dicots is organized in three clonally distinct cell layers (L1, L2 and L3). Cells in the two outer layers (L1 and L2) are characterized by anticlinal divisions, while cells in the L3 layer divide in all planes. The outermost L1 layer gives rise to the epidermis, while the subepidermal and vascular tissues of aerial organs are the result of mitotic activity in L2 and L3 layers [30,36,37].

In the process of de novo shoot organogenesis, after acquiring the organogenic competence, the pluripotent primordia induced on CIM begin to acquire the morphological and functional characteristics of the shoot meristem. As already mentioned, the last stage of the de novo shoot organogenesis is the formation of the SAM, whereby the expression of *WUS* and *STM* plays a central role. *WUS* is involved in the specification and maintenance of stem cells in the shoot meristem and is necessary for the establishment of the shoot stem cell niche, while *STM* stimulates cell proliferation and prevents their differentiation. These genes are positively regulated by cytokinin, and the course of their expression coincides with the transfer of explants from the CIM to the SIM, with *WUS* being expressed slightly earlier than *STM* [38]. The SAM is zoned spatiotemporally, requiring the maintenance of morphogenic cell-to-cell signaling systems governed by either cytokinin or auxin signals that regulate cell-specific transcription patterns in order to maintain a correct balance between cell division and differentiation. During the development of the shoot meristem, the expression of *WUS* is initially observed in the surrounding area of the parts that represent shoot precursors, where *CUP-SHAPED COTYLEDON2* (*CUC2*) is expressed (Figure 1). Thereafter, *CUC2* is slowly silenced under the influence of a strong cytokinin signal, giving “space” to *WUS* to start inducing the respecification of cells into shoot meristem cells [39].

*WUS* is expressed in the organizing center of the meristem, and the WUS morphogen migrates through the plasmodesmata acropetally, i.e., to the central zone where it binds to the promoter of *CLAVATA3* (*CLV3*), inducing its expression (Figure 1). CLV3 is a small extracellular peptide that represses *WUS* expression outside the OC through a negative feedback loop (Figure 1), resulting in the maintenance of a constant number of stem cells spatially confined to the OC [31,32,35]. The CLV3-mediated repression of *WUS* was shown to occur through two distinct pathways. One of them is localized to the CZ and relies on CLV3 binding to the CLV1 receptor kinase. The second pathway, which appears to function throughout the SAM, involves CLV2 as the receptor for the CLV3 ligand; however, since CLV2 lacks a cytosolic kinase domain, its function relies on the association with the receptor kinase CORYNE (CRN) [40,41].

Recently, a novel regulatory circuit has been discovered, involving a mutually repressive relationship between *WUS* and its paralog, *WOX13* [42]. As an important regulator of tissue repair and organ reconnection, *WOX13* is regulated by auxin and is responsible for the maintenance of callus through the cell fate specification of highly expanded cells that constitute the disorganized callus tissue; the callus is also maintained through a *WOX13*-mediated repression of *WUS*. Thus, a shift in the balance between *WOX13* and *WUS* expression, initiated by cytokinin upon its uptake from the SIM, is needed to enable the transition from the callus to the shoot identity acquisition [42]. When a *WUS*-expressing domain is established in the shoot promeristem, it maintains its own expression by promoting cytokinin signaling through the repression of the negative regulators of the cytokinin response, *ARABIDOPSIS RESPONSE REGULATOR7* (*ARR7*) and *ARR15* [43] (Figure 1).

Thus, cytokinin plays a central role in the establishment and regulation of shoot meristem activity. In tobacco (*Nicotiana tabacum* L.), an exogenous supplementation of the cytokinin benzyladenine in concentrations up to 10 μM positively affected both the SAM size and the number of cells in the L1 layer of CZ [44]. Furthermore, analyses of cytokinin-deficient *Arabidopsis* mutants and lines overexpressing enzymes involved in cytokinin degradation showed a reduced size of the shoot apical meristem [35]. In addition, the *AHK4* receptor gene is expressed in the center of the SAM, overlapping with the OC in its distal part and positively regulating *WUS* expression [35]. Interestingly, *WUS* acts as a regulator of cell differentiation in the PZ besides its obvious key function in the maintenance of pluripotent stem cells in the CZ. Accordingly, it is observed that the transient downregulation of *WUS* leads to an increase in the number of auxin-responsive cells within the PZ. It is known that the control of auxin levels and suppression of differentiation have to be maintained for proper SAM function; therefore, this is an example of how the positive regulation of *WUS* expression affects auxin signaling in order to prevent premature cell differentiation [45,46].

*SHOOTMERISTEMLESS* (*STM*) is a member of the *KNOTTED1-LIKE HOMEOBOX* gene family, and it was originally thought to be a key factor in the formation and maintenance of the SAM, since after the induction of *STM* expression, the process of organogenesis occurs independently of incubation on SIM [14]. The activity of *STM* is closely related to transcription factors CUC1 and CUC2 which stimulate it, while STM represses *CUC1* and *CUC2*, indicating the existence of a negative feedback loop between *STM* and *CUC1*/*2* (Figure 1). The vital role of *STM* in maintaining meristem activity and preventing cell differentiation was also corroborated by the finding that these functions are disabled in *stm* mutants [47]. Like *WUS*, *STM* also affects cytokinin homeostasis by stimulating the transcription of the cytokinin biosynthesis gene *ISOPENTENYL-TRANSFERASE7* (*IPT7*), while *STM* itself is positively regulated by cytokinins (Figure 1), thus establishing a positive feedback loop between cytokinins and *STM* [48]. It was observed that *STM* is required for the activation of *WUS* expression. When *stm* mutants of *Arabidopsis* are compared to wild-type plants, the transcript levels of *WUS* fell to 40–60% of WT *WUS* transcript levels. This decrease in *WUS* transcription is detected before any impairments in shoot development associated with the lack of *STM* expression could be observed at the morphological level [49].

Gordon and co-authors [39] examined the shoot organogenesis of partially deficient *wus* mutants and demonstrated that regeneration proceeds unhindered as soon as *STM* expression is switched on. Thus, *STM* appears to be crucial for the acquisition of shoot identity. However, in current models of shoot regeneration, the roles of both *STM* and *WUS* are typically acknowledged as very important. Both genes are importantly involved in the formation of the SAM; they are stimulated by cytokinin, they act independently of each other and their functions are complementary [14]. Although they induce the expression of different downstream target genes, it seems that the co-expression of *STM* and *WUS* specifically, and subsequently the expression of all their target genes, results in a synergistic effect in suppressing cell differentiation, and the specification and maintenance of the shoot stem cell niche [50].

## 3. The Cytokinin Signaling Cascade

**Figure 2:**
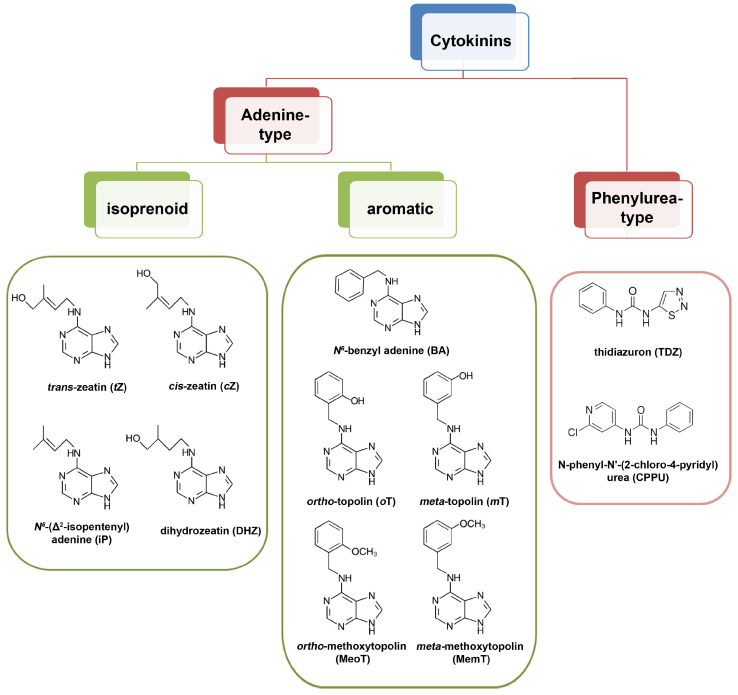
Main types of bioactive cytokinin molecules. Naturally occurring (adenine-type) cytokinins are divided into isoprenoid and aromatic cytokinins, depending on the structural type of their side chain. The adenine-type bioactive cytokinins can undergo conjugation to form various types of cytokinin conjugates (for details, see [52]). Synthetic substances with cytokinin activity have a phenylurea-type structure, the two most commonly used in plant biotechnology being thidiazuron (TDZ) and *N*-phenyl-*N*′-(2-chloro-4-pyridyl)urea (CPPU).

Cytokinins are a group of plant hormones that affect a wide range of developmental and physiological processes, including the stimulation of cell divisions, regulation of meristem cells, shoot development, senescence, apical dominance, chloroplast formation, etc. [51]. Naturally occurring cytokinins are derivatives of *N*^6^-substituted adenine, and based on the chemical properties of the substituent, they are divided into isoprenoid and aromatic cytokinins [52]. Conversely, synthetic cytokinins comprise derivatives of phenylurea, such as thidiazuron (TDZ) and *N*-phenyl-*N*′-(2-chloro-4-pyridyl)urea (CPPU) [53] (Figure 2). Different types of natural and synthetic cytokinins bind to different cytokinin receptors with differential affinities [54]. All types of cytokinin—isoprenoid, aromatic, and phenylurea type, are commonly used as active components of the SIM in protocols for in vitro shoot regeneration [25]. In model systems such as kohlrabi, isporenoid-, aromatic- and phenylurea-type cytokinins have shown distinct responses in terms of dynamics and efficiency of callus formation and shoot organogenesis, which has been attributed to differential effects on both the metabolism of all endogenous phytohormones [26] and the expression of particular organogenesis-related genes, including the genes involved in cytokinin signaling [27].

**Figure 3:**
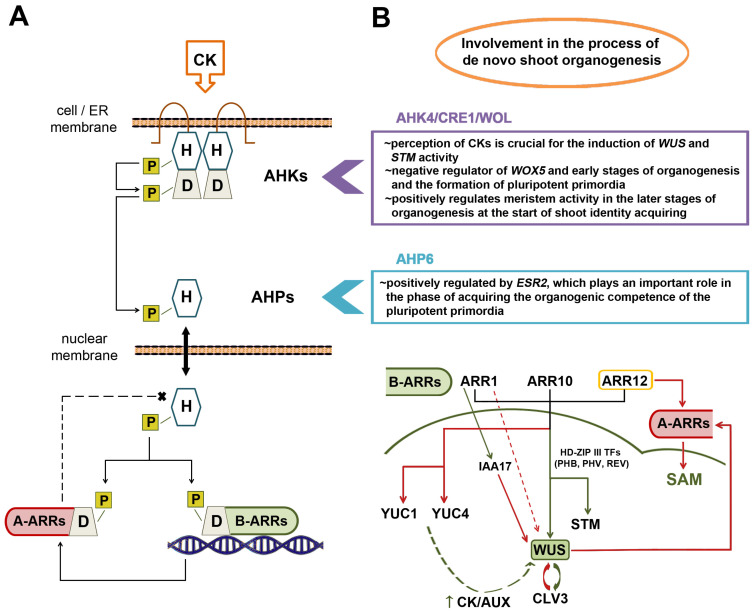
The cytokinin signaling network in *Arabidopsis*. (**A**) Cytokinin (CK) binds to the receptor ARABIDOPSIS HISTIDINE KINASE (AHK) domain on the outside of the plasma membrane or endoplasmic reticulum (ER), thereby inducing the activity of the histidine–kinase domain (H), from which the phosphate group (P) is transferred to the aspartate residue of the receptor domain (D). The phosphate group (P) is further transferred to the His residue of ARABIDOPSIS HISTIDINE PHOSPHOTRANSFER PROTEINS (AHPs). These proteins constantly shuttle between the cytosol and the nucleus, where they are able to further phosphorylate the ARABIDOPSIS CYTOKININ RESPONSE REGULATORs (type-A and -B ARRs), which are activated upon receiving a phosphate group, further regulating the target genes of CK action. (**B**) The implicated role of particular elements of the CK signaling cascade during the de novo shoot organogenesis and formation of shoot apical meristem (SAM). Green arrows mark the upregulation and red arrows mark downregulation of gene/protein activity. Full arrows indicate direct regulation and dotted arrows indicate indirect regulation. TF—transcription factor, AUX—auxin.

Cytokinin perception initiates a phosphorelay signaling cascade similar to bacterial two-component signaling systems, which involves the transfer of a phosphate residue between histidine (His) and aspartate (Asp) residues of proteins that represent elements of the cytokinin signaling cascade [55] (Figure 3).

In the model plant *A. thaliana*, three transmembrane receptors (homodimers by structure, when they are in their activated form) which can bind cytokinins have been described: ARABIDOPSIS HISTIDINE KINASE2 (AHK2), AHK3 and AHK4/CRE1/WOL [56]. In *Arabidopsis*, these cytokinin receptors are located predominantly in the endoplasmic reticulum [57]. However, since compelling evidence argues for cytokinin signaling both from the endoplasmic reticulum and the plasma membrane, it is likely that both pathways play distinct roles in plant growth and development [58,59,60]. All three cytokinin receptors possess conserved N-terminal extracellular CHASE (cyclase/His-kinase-associated sensing extracellular) domains to which the cytokinin binds, thereby inducing autophosphorylation of the His–kinase in the cytoplasmic domain of the receptor [56]. The next step includes phosphate transfer to the Asp residue of the receiver receptor domain (Figure 3A).

This step is followed by ARABIDOPSIS HISTIDINE PHOSPHOTRANSFER PROTEINS (AHPs), as the next elements of signal transduction downstream of the receptor, containing a conserved His residue to which the phosphate group can be transferred from the Asp residue of the cytosolic domain of AHK. This transfer of the phosphate group is required for the activation of AHP as part of the signaling cascade (Figure 3A). The AHP family of *Arabidopsis* includes six AHP proteins, five of which represent “true” AHP proteins (AHP1-AHP5) with a conserved His residue for phosphotransfer, as well as a pseudo-AHP (APHP1/AHP6), in which the His residue is replaced with Asn [61]. Analyses of single *ahp* mutants did not show altered sensitivity to cytokinins and thus had no effect on growth and development, while some double *ahp* mutants showed increased resistance to cytokinin, indicating partial functional redundancy within the AHP family. Thus, AHP proteins function as positive regulators of cytokinin signaling [62]. The AHP proteins continuously shuttle between the cytosol and the nucleus independently of the existence of cytokinin signals [63]. This allows for the transmission of cytokinin signals to the nucleus, where phosphorylated AHPs are able to act downstream by phosphorylating a conserved Asp residue in the receiver domain of type-B and type-A ARABIDOPSIS RESPONSE REGULATOR (ARR) proteins, which are positive and negative regulators of the cytokinin response, respectively (Figure 3A) [55].

The AHP6 protein, however, does not participate in the cytokinin signaling cascade because it has no phosphotransfer activity; it functions, instead, as an inhibitor of the cytokinin signal. Mähönen and co-authors [64] experimentally confirmed that AHP6 can interfere with the phosphorelay machinery by blocking the transfer of the phosphate group between AHP1 and ARR1 proteins. Alongside that, a loss-of-function *ahp6* mutation could partially restore the cytokinin signal in *wooden leg* (*wol*) mutants that contain a loss-of-function mutation in the cytokinin receptor gene *AHK4* [64].

Downstream of the AHPs, the type-B ARR proteins play a central role in the initial transcriptional response to cytokinin. They represent transcriptional activators that regulate the expression of cytokinin signaling effectors, including type-A ARR proteins (Figure 3A). Eleven type-B proteins have been described in *A*. *thaliana*, at least six of which are positive regulators of the cytokinin response [65]. Genetic analyses suggested that ARR1, ARR10 and ARR12 play the most significant role in the regulation of transcriptional and physiological responses to cytokinins [66].

Conversely, ten type-A ARR genes have been described in *A*. *thaliana* and were originally defined as primary cytokinin response genes [67,68]. Their protein products are negative regulators of the cytokinin response that act on other elements of cytokinin signaling through a negative feedback mechanism (Figure 3A), and in addition, they participate in the regulation of the circadian rhythm and phytochrome function [69]. Unlike type-B ARRs, type-A ARR regulators lack a DNA-binding domain; thus, the exact molecular mechanisms of their repressive action remain elusive to date [55]. It has also been proven that these regulators negatively affect shoot meristem formation [43,70]. In response to the cytokinin signal, type-A ARR proteins are directly transcriptionally induced by the activity of type-B ARR proteins (Figure 3A). Type-A ARR proteins perform their physiological functions when they are phosphorylated, whereas unphosphorylated type-A ARRs are targeted for degradation [55].

## 4. The Roles of the Cytokinin Signaling Cascade Elements in De Novo Shoot Organogenesis and Shoot Apical Meristem Induction

The cytokinin signaling cascade includes four distinct signaling modules: receptors/histidine kinases (AHKs), histidine phosphotransfer proteins (AHPs), and type-B and type-A cytokinin response regulators (ARRs). As exogenous cytokinin affects the change in the morphogenic course of primordia following the transfer of explants from the CIM to the SIM, it is implied that cytokinin signal transduction and the included elements are a prerequisite for further steps in organogenesis. In this context, the regulation of de novo shoot organogenesis can be viewed in light of the action of different modules of the cytokinin signaling cascade.

### 4.1. The Roles of Cytokinin Receptors—Histidine Kinases (AHKs)

Current evidence argues for an extensive involvement of AHK4/CRE1/WOL, but not AHK2 and AHK3, in the process of de novo shoot organogenesis (Figure 3B). When shoot regeneration was investigated in double *ahk2ahk3*, *ahk2ahk4* and *ahk3ahk4* mutants of *A. thaliana*, it was reported that the regeneration phenotypes of both *ahk2ahk4* and *ahk3ahk4* greatly differed from *ahk2ahk3*. Namely, shoot regeneration was significantly disturbed in *ahk2ahk4* and *ahk3ahk4* mutants, but much less so in double *ahk2ahk3* mutants, since the explants containing a loss-of-function mutation in *AHK4* could not regenerate shoots [71].

On the other hand, the earlier stages of organogenesis were more efficiently induced in *ahk4* mutants than in wild-type plants, which led to the assumption that AHK4 negatively regulates the early steps of de novo organogenesis, such as primordium formation. The AHK4 receptor also positively regulates meristem activity in the later stages of organogenesis when the primordium begins to acquire shoot identity. Additionally, Pernisová and co-authors [71] reported that the *ahk4*, *ahk2ahk4* and *ahk3ahk4* mutants exhibited an increased expression of *WOX5*, a key gene for maintaining the root-like stem cell niche of the pluripotent primordium, while in the double *ahk2ahk3* mutants, *WOX5* was downregulated. Thus, cytokinin signaling through the AHK4 receptor negatively regulates *WOX5* gene expression, consequently inhibiting the initiation of primordia (Figure 3B). Importantly, the perception of cytokinins by the receptor AHK4/CRE1/WOL is crucial for the induction of *WUS* and *STM* activity in the final phase of shoot regeneration [12].

### 4.2. Histidine Phosphotransfer Proteins (AHPs)

As for histidine phosphotransfer proteins (AHPs), there is still no literature data clarifying how these proteins participate in the regulation of de novo shoot organogenesis and shoot apical meristem activity. It was experimentally confirmed that ENHANCER OF SHOOT REGENERATION2 (ESR2), which plays an important role in the phase of the acquisition of organogenic competence of the pluripotent primordia, positively regulates the *AHP6* gene encoding the pseudo-AHP protein AHP6 (Figure 3B), but it has not yet been revealed whether and how such upregulation affects the efficiency of shoot regeneration [72]. To date, no other published research has addressed the role of AHPs in shoot regeneration.

### 4.3. Type-B Response Regulators (ARRs)—Positive Regulators of De Novo Organogenesis and SAM Activity

Cytokinin response regulators that were first identified in *Arabidopsis* and hence named ARABIDOPSIS RESPONSE REGULATORS (ARRs) represent the final component in the cytokinin signaling cascade, enabling the conversion of the cytokinin signal into a precise molecular instruction resulting in a specific physiological response. Thus, the formation and maintenance of shoot meristems without the activity of these response regulators is impaired.

In *A. thaliana*, four type-B ARR regulators have been described that play key roles in the positive regulation of *WUS* during SAM formation: ARR1, ARR2, ARR10 and ARR12 [73]. The cytokinin taken up from regeneration media induces histone demethylation within the *WUS* locus, after which the ARR regulators activate three HD-ZIP III transcription factors: PHABULOSA, PHAVOLUTA and REVOLUTA (PHB, PHV and REV, respectively) which induce *WUS* transcription and also positively regulate *STM*, which, in addition to *WUS*, is the most important regulator of SAM formation (Figure 3B). Triple *phbphvrev* mutants were heavily impaired in the formation of shoot apical meristem [73]. An induction of *WUS* expression does not necessarily occur through the three HD-ZIP III transcription factors, since type-B ARR regulators can also activate *WUS* by binding directly to its promoter [74]. Compared with the wild-type phenotype, *arr1arr10arr12* and *arr2arr12* mutants showed reduced shoot regeneration efficiency, and transcriptional analysis of triple *arr1arr10arr12* mutants showed the absence of *WUS* and *CLV3* transcripts [73]. Furthermore, by monitoring the spatiotemporal patterns of *ARR1*, *ARR10* and *ARR12* expression, it was concluded that these genes are expressed locally and that during the incubation of *A. thaliana* explants on the SIM, ARR signals were limited to discrete regions in the central part of the shoot meristem that overlap with the *WUS*-expressing domain [74]. In addition, the inducible silencing of *ARR1*, *ARR10* and *ARR12* severely reduced shoot regeneration, with root regeneration occurring instead, while the analysis of double *arr1arr10*, *arr1arr12* and *arr10arr12* mutants showed an obvious reduction in *WUS* transcripts; thus *ARR1*, *ARR10* and *ARR12* were concluded to positively regulate *WUS* transcription. Interestingly, ARR1, ARR10 and ARR12 were shown to be bifunctional transcription factors that play dual roles in the regulation of *WUS*. Besides activating *WUS* transcription, they also negatively regulate the expression of auxin biosynthesis genes *YUC1* and *YUC4* in the SAM domain where *WUS* is expressed (Figure 3B). Consequently, a high ratio of cytokinin-to-auxin is ensured in this part of the meristem in order to maintain a high level of *WUS* expression [74]. On the other hand, Dai and co-authors [75] confirmed the essential role of ARR12 in the regulation of de novo SAM formation, whereby it directly binds to *WUS* promoters and activates its expression. They showed that an increased expression of *ARR12* enhances shoot regeneration efficiency, whereas the loss-of-function *arr12* mutants almost completely lost the ability to regenerate shoots. In *arr12* mutants, an increase was also observed in the expression of *ARR5*, *ARR7*, *ARR9* and *ARR15*, which encode type-A ARR regulators that are negative regulators of cytokinin signaling and act as repressors of shoot regeneration. The upregulation of *ARR5*, *ARR7*, *ARR9* and *ARR15* in *arr12* mutants implies that ARR12 is an essential negative regulator of these genes and that the decrease in *WUS* expression level is not exclusively caused by the lack of binding of ARR12 to *WUS* promoters, but also by the repressive action of type-A ARRs. The expression level of genes essential for de novo shoot regeneration and SAM formation, *WUS*, *CLV3*, *STM* and *CUC2*, was significantly reduced in *arr12* mutants, while conversely, in explants overexpressing *ARR12*, an increase in the expression of these morphogenes was recorded [75]. In addition, the binding sites for ARR10 were also discovered in the *WUS* promoter sequence, and upregulation of *ARR10* resulted in the activation of *WUS* transcription and increased shoot regeneration efficiency [76].

By analyzing the transcriptional network based on the action of type-B regulators, it was shown that they bind alternatively to the promoters of different genes [77]. As a result, it was found that the gene promoters to which B-ARR proteins bind are rich in *cis* regulatory elements 5′-AGATHY-3′ (H = A/T/C; Y = T/C). B-ARR proteins are not regulated by cytokinin at the transcriptional level but are subject to post-translational modifications, and one of the possible reasons for this is that B-ARR proteins would bind “loosely” to the promoters of target genes if they did not undergo post-translational modifications. ARR1 showed the highest degree of cytokinin-dependent binding to promoters of target genes [77]. Without cytokinin treatment, ARR1 recognized 2815 potential target genes, while after 4 h of cytokinin treatment, this number almost doubled (5128), and after 3 days, the number increased to 10,340. These results suggest that prolonged cytokinin treatment can change the chromatin structure so that a greater number of ARR1-binding sites in gene promoters would become available [77].

Type-B ARRs are all generally considered to be positive regulators of the cytokinin response and thus positively regulate de novo shoot regeneration. However, recently, it was discovered that a type-B regulator, ARR1, actually exerts an inhibitory effect on the induction of de novo shoot regeneration by ARR12 [78] (Figure 3B). It was shown that *arr1* mutants displayed an increase in shoot regeneration efficiency when incubated on SIM, compared to the wild-type plants. In addition to inhibiting SIM-induced de novo shoot regeneration, ARR1 also inhibits CIM-induced callus formation, so it is hypothesized that ARR1 inhibits the process of de novo shoot regeneration by limiting the formation of cells competent for shoot development. In addition, ARR1, together with ARR12, performs a complex regulation of *WUS* and *CLV3*. ARR12 functions as a positive regulator of *CLV3* transcription, while ARR1 acts as an inhibitor of *CLV3* transcription induced by ARR12 by competing for the same binding site on the promoter of *CLV3*, partially silencing the positive regulatory effect of ARR12 on *CLV3*. Also, ARR1 competes with ARR12 for binding sites on *WUS* promoter sequences, delaying the establishment of the intercellular gradient of WUS transcripts in the SAM, which also has an inhibitory effect on de novo shoot regeneration. Another indirect inhibitory effect of ARR1 on shoot regeneration is manifested through the transcriptional activation of *IAA17*, an auxin-responsive gene that functions as a transcriptional inhibitor of *WUS* [78]. Thus, it can be construed that ARR10 and ARR12 represent factors of essential importance for the positive regulation of shoot apical meristem function, while ARR1 serves to fine-tune their response by “badly playing its role” as a type-B regulator. This subtle functional differentiation between individual members of the type-B ARR family suggests a novel dimension to the concept of genetic functional redundancy: although ARR1, ARR10 and ARR12 have been demonstrated to perform functionally redundant roles in multiple physiological processes such as, for instance, salt tolerance [79] or gynoecium development [80], future insight into fine details of their regulatory roles may reveal subtle, unexpected individual differences that enable the fine-tuning of these or other redundantly regulated physiological processes [12].

### 4.4. Type-A Response Regulators (ARRs)—Negative Regulators of De Novo Organogenesis and SAM Activity

In addition to type-B ARRs, the negative regulators of the cytokinin response, type-A ARRs, have also been the subject of research in the context of their regulatory influence on de novo organogenesis and shoot apical meristem formation.

Type-A ARRs have a negative effect on meristem size and are directly silenced by WUS, thus maintaining normal meristem activity (Figure 3B). WUS represses the closely related *ARR5*, *ARR6*, *ARR7* and *ARR15* genes, abolishing their repressive influence on the maintenance of the SAM [43]. In addition, ARR7 and ARR15 are strong inhibitors of de novo shoot regeneration, and accordingly, explants of *A. thaliana* plants overexpressing *ARR7* and *ARR15* exhibited reduced shoot regeneration efficiency, while conversely, loss-of-function *arr7* and *arr15* mutants showed enhanced callus formation and shoot regeneration [70]. The function of ARR7 and ARR15 in inhibiting shoot regeneration seems to be more important than that of ARR5 and ARR6, since the double loss-of-function *arr5arr6* mutants showed a lesser increase in shoot regeneration efficiency compared to single *arr7* or *arr15* mutants [70].

Che and co-authors [81] showed that the expression of *ARR5* is positively regulated during the incubation of *Arabidopsis* explants on the CIM, and an increase in expression was also detected after the transfer of explants to the SIM, where the maximum expression was reached on the sixth day. After that, the expression level started to decrease, which coincided with the beginning of shoot formation, suggesting that *ARR5* was silenced by WUS. A similar two-phase upregulation of *ARR5* was observed during one-step shoot regeneration from intact seedlings of kohlrabi (*Brassica oleracea* var. *gongylodes*) [27]. The first *ARR5* upregulation peak occurred soon after transfer to the SIM, presumably as an immediate result of the uptake of the cytokinin from the media and subsequently induced transcriptional upregulation, after which the appearance of the first calli corresponded to the subsequent downregulation of *ARR5*. However, the second peak was recorded after about three weeks, accompanied with a sharp decrease in expression overlapping with shoot development, confirming that a common expression profile of *ARR5* is shared between the two-step shoot regeneration from *Arabidopsis* root explants and the one-step shoot regeneration from kohlrabi seedlings [27].

Overall, the WUS/ARR-A system functions as a negative feedback loop, with WUS repressing the transcription of type-A *ARR* genes that have a repressive effect on SAM activity, thereby positively regulating cytokinin signaling, while cytokinin signaling, in turn, positively regulates type-A *ARR* genes in order to finely modulate meristem activity (Figure 3B).

## 5. Conclusions

Owing to its vast biotechnological, industrial and conservational potential, de novo shoot organogenesis has been the subject of research for decades but has remained a great scientific challenge both in the theoretical sense and the applicative context. The process of de novo organogenesis is based on the cooperative action of the phytohormones cytokinin and auxin and their mutual balance, as well as the presence of stem cells, capable of differentiating, forming plant tissues and, finally, organs. Thanks to accumulated evidence, the molecular mechanisms of de novo shoot organogenesis have been explained in detail. However, to date, the focus of research has mostly been directed toward developmental molecular processes and the main genes that determine the further morphogenic flow and fate of the cells. Little is still known about the mechanisms of uptake of phytohormones from the regeneration media and their transport to target cells. There is a wide array of environmental factors (light, temperature, air humidity, culture vessel aeration, etc.) that significantly affect the process of de novo organogenesis, but they have been largely neglected so far.

Moreover, although the underlying genetic regulatory networks have been determined to some extent, certain details about the cytokinin-mediated regulation of shoot regeneration remain very poorly explained. For instance, whether the keystone events in shoot regeneration depend on cytokinin signals from the endoplasmic reticulum, or from the plasma membrane remains practically unknown. In addition, only during the last few years has more attention been paid to the molecular mechanisms of the cytokinin signaling cascade elements, especially the AHP proteins and ARR regulators of the cytokinin response, which represent the key link between the cytokinin uptake from the medium and the resulting shoot regeneration. Individual differences between seemingly redundant members of response regulator protein families are starting to emerge, suggesting distinct regulatory functions that, besides classical stimulatory and inhibitory roles, encompass more complex and sophisticated functional identities, such as “players you don’t want on your team”, as is the case of ARR1.

The hormonal and genetic regulation of de novo shoot regeneration is extremely intricate and complex, and as such, past attempts at clarifying it have heavily relied on simplifications. These simplifications were historically needed to help us grasp the basic principles on which shoot regeneration works; however, in order to improve the efficiency and expand the applications of current biotechnological practices, we will need an even deeper and more thorough understanding of the particular details of the regulatory networks that underlie this complex developmental program. In order to do that, we need to dissect, challenge and re-evaluate our dogmatic understanding of the principles of phytohormone action and shoot regeneration. Since phytohormones are the central regulators of plant growth and development, a more comprehensive and detailed understanding of the roles of the cytokinin signaling network in shoot regeneration can be considered a good start.
